# The long non-coding RNA HOTTIP enhances pancreatic cancer cell proliferation, survival and migration

**DOI:** 10.18632/oncotarget.3450

**Published:** 2015-03-25

**Authors:** Yating Cheng, Indira Jutooru, Gayathri Chadalapaka, J. Christopher Corton, Stephen Safe

**Affiliations:** ^1^ Department of Veterinary Physiology and Pharmacology, Texas A&M University, 4466 TAMU, College Station, TX 77843-4466, USA; ^2^ Institute of Biosciences and Technology, Texas A&M Health Science Center, Houston, TX 77030–3303, USA; ^3^ Integrated Systems Toxicology Division, US-EPA, MD B143-06, Research Triangle Park, NC 27711, USA; ^4^ Covance, Inc., Madison, WI 53704, USA

**Keywords:** HOTTIP, lncRNA, HOX genes, pro-oncogenic, HOTAIR

## Abstract

HOTTIP is a long non-coding RNA (lncRNA) transcribed from the 5′ tip of the HOXA locus and is associated with the polycomb repressor complex 2 (PRC2) and WD repeat containing protein 5 (WDR5)/mixed lineage leukemia 1 (MLL1) chromatin modifying complexes. HOTTIP is expressed in pancreatic cancer cell lines and knockdown of HOTTIP by RNA interference (siHOTTIP) in Panc1 pancreatic cancer cells decreased proliferation, induced apoptosis and decreased migration. In Panc1 cells transfected with siHOTTIP, there was a decrease in expression of 757 genes and increased expression of 514 genes, and a limited gene analysis indicated that HOTTIP regulation of genes is complex. For example, Aurora kinase A, an important regulator of cell growth, is coregulated by MLL and not WDR5 and, in contrast to previous studies in liver cancer cells, HOTTIP does not regulate HOXA13 but plays a role in regulation of several other *HOX* genes including *HOXA10*, *HOXB2*, *HOXA11*, *HOXA9* and *HOXA1*. Although HOTTIP and the HOX-associated lncRNA HOTAIR have similar pro-oncogenic functions, they regulate strikingly different sets of genes in Panc1 cells and in pancreatic tumors.

## INTRODUCTION

Long non-coding RNAs (lncRNAs) are defined as transcripts containing >200 nucleotides and are typically transcribed by RNA polymerase II [1]. Although the existence of lncRNAs has been known for several decades, it is only in the last ten years that the multiple functions of the lncRNA components of the noncoding genome have been determined. LncRNAs play important roles in maintaining cellular homeostasis during cell/tissue development and they are also critical factors in pathophysiology including cancer [1–6]. The molecular modes of action of lncRNAs are highly variable and include their functions as molecular scaffolds for stabilizing protein-protein and protein-DNA interactions; they can also act as decoys and guides that facilitate both proximal and distal macromolecular interactions which are usually on a genome template [2, 3].

LncRNAs have been investigated in tumors and cancer cells derived from multiple sites, and there is strong evidence that their overexpression or underexpression can influence cancer cell growth, survival and migration/invasion [7–9]. HOX transcript antisense RNA (HOTAIR) is a 2.2 kb lncRNA in the mammalian *HOXC* locus that serves as sequence-specific scaffold for at least two histone modification complexes, namely polycomb repressive complex (PRC2) and the LSD1/CoREST/REST complex [7–9]. In tumors and cancer cells, HOTAIR interactions with these histone modification complexes modulate expression of tumor type-dependent gene sets, and knockdown or overexpression studies show that HOTAIR is an important pro-oncogenic factor that plays a role in cancer cell proliferation, survival and migration/invasion [8–14]. HOTAIR is also a tumor-specific negative prognostic factor for the survival of cancer patients and can be detected in serum [13].

HOXA transcript at the distal tip (HOTTIP) is another HOX-associated lncRNA transcribed from the 5′ tip of the HOXA locus, and HOTTIP is associated with the PRC2 and WDR5/MLL1 chromatin modifying complexes and directly binds WDR5 [15]. HOTTIP primarily coordinates expression of genes associated the HOXA locus in fibroblasts [15], and a recent paper showed a close association between HOTTIP and HOXA13 in hepatocellular carcinomas (HCCs) [16]. For example, both HOTTIP and HOXA13 are upregulated in HCCs and are associated with metastasis and decreased patient survival [16]; moreover, individual knockdown of HOTTIP or HOXA13 by RNA interference (RNAi) in HCC cell lines results in downregulation of HOXA13 and HOTTIP, respectively. Moreover, RNAi studies showed that knockdown of HOTTIP and HOXA13 decreased cell proliferation but did not affect apoptosis in HCC cells [16].

Previous studies in this laboratory showed that knockdown of HOTAIR in pancreatic cancer cells decreased proliferation, induced apoptosis, and inhibited invasion, and this was associated with changes in expression of genes associated with these pathways [12]. We have now investigated the role of HOTTIP in pancreatic cancer cells and have observed pro-oncogenic functions similar to that reported for HOTAIR, even though both lncRNAs elicit their effects by regulating expression of different sets of genes by different pathways.

## RESULTS

### HOTTIP: functional studies as determined by knockdown and overexpression

**Figure 1:**
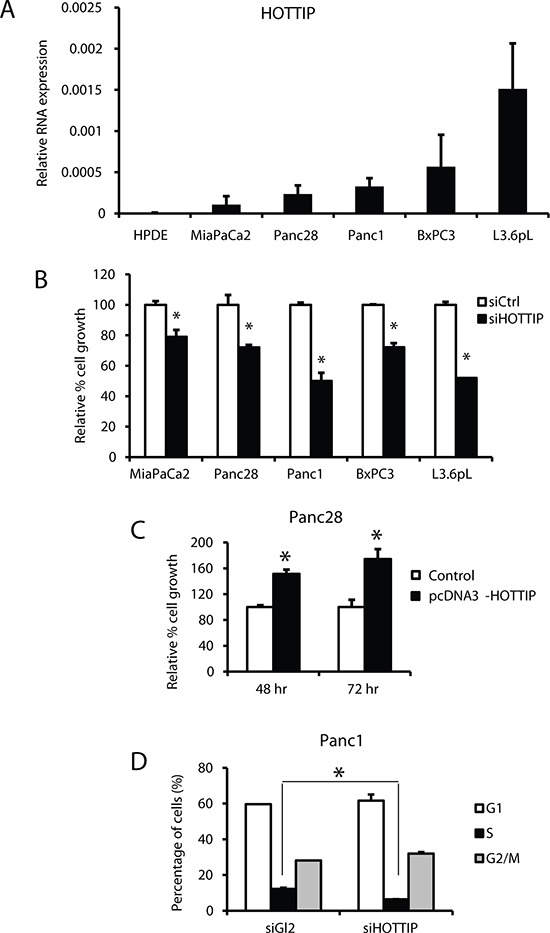
Effects of HOTTIP in pancreatic cell proliferation and cell cycle **(A)** HOTTIP expression relative to the housekeeping gene TATA-binding protein (TBP) was determined by real time PCR as described in the Materials and Methods. HOTTIP knockdown by RNAi knockdown in Panc28, MiaPaCa2, Panc1, BxPC3 and L3.6pL cells inhibited cell growth **(B)**, whereas HOTTIP overexpression in Panc28 cells promoted cell proliferation **(C)**. **(D)** The effect of siHOTTIP (knockdown) on cell cycle progression in Panc1 cells was determined by FACS analysis as described in the Materials and Methods. Results (A–D) are expressed as means ± SD for 3 replicates. Cells transfected with a non-specific oligonucleotide (siCtrl) were used as controls and significant (*p* < 0.05) changes are indicated (*).

**Figure 2:**
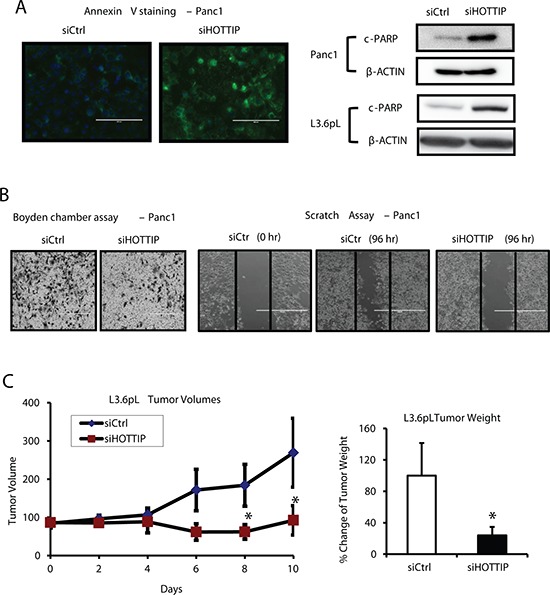
Effects of HOTTIP in pancreatic cancer cell apoptosis, migration and tumor growth **(A)** Panc1 cells were transfected with siHOTTIP and after 48 hr, and the increase in Annexin V staining and induction of PARP cleavage were determined by fluorescence and Western blots analysis respectively. **(B)** HOTTIP knockdown reduced cell migration as determined by the Boyden chamber and scratch assay as discussed in the Materials and Methods. **(C)** HOTTIP was silenced in L3.6pL cells which were then used in the athymic nude mice as xenografts. Tumor volumes were determined for up to 10 days, and tumor weights were measured after the animals were sacrificed at Day 10. Cells transfected with a non-specific oligonucleotide (siCtrl) were used as controls, and five mice were used in each treatment group. Significant (*p* < 0.05) changes are indicated (*).

Figure 1A illustrates the expression of HOTTIP in five pancreatic cancer cell lines in which high expression is observed in Panc1, L3.6pL and MiaPaCa2 cells and lower (> 2-fold) expression in Panc28 and BxPC3 cells. HPDE cells are non-transformed immortalized pancreatic epithelial cells and only minimal expression of HOTTIP was observed. Knockdown of HOTTIP by RNAi significantly decreased proliferation of Panc1, L3.6pL, Panc28, BxPC3 and MiaPaCa2 cells (Figure 1B) and overexpression of HOTTIP increased proliferation of Panc28 cells (Figure 1C), and the growth promoting effects of HOTTIP were similar to those previously reported for HOTAIR in pancreatic cancer cells [12]. Knockdown of HOTTIP in Panc1 cells slightly decreased the percentage of cells in S phase and increased the percentage of cells in G_2_/M phase compared to Panc1 cells transfected with the scrambled siRNA (Figure 1D). Knockdown of HOTTIP by RNAi induced Annexin V staining and enhanced PARP cleavage in Panc1 cells (Figure 2A), demonstrating that HOTTIP plays a role in pancreatic cancer cell survival. Moreover, results of Boyden chamber and scratch assays (Figure 2B) show that knockdown of HOTTIP significantly decreased Panc1 cell migration and these results were similar to those previously observed in comparable experiments with HOTAIR in pancreatic cancer cells [12]. Moreover, like HOTAIR, knockdown of HOTTIP in L3.6pL cells which were used in a xenograft model in athymic nude mice decreased tumor growth and tumor weights (Figure 2C) compared to tumors in cells expressing HOTTIP (transfected with a non-specific oligonucleotide). Thus, HOTTIP plays a pro-oncogenic role in pancreatic cancer cells.

### Regulation of gene expression by HOTTIP determined by knockdown and analysis by beadchip arrays

**Figure 3:**
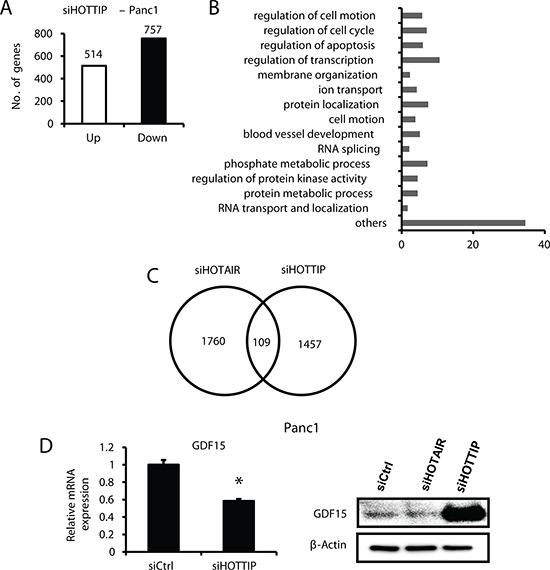
Gene regulation by HOTTIP and compared to HOTAIR in Panc1 cells **(A)** Panc1 cells were transfected with siHOTTIP or siCtrl and gene expression was analyzed using HumanHT-12 v4 expression beadchip (Illumina Inc.) array, and **(B)** the effects of siHOTTIP on different pathways were determined by gene ontology enrichment analysis. **(C)** Overlap of common genes was observed after treatment of Panc1 cells with siHOTTIP or siHOTAIR. **(D)** Panc1 cells were transfected with siHOTTIP and GDF15 mRNA, and protein levels were determined compared to the effects of siHOTAIR. Results for GDF15 mRNA are means ± SD for 3 replicated determinations, and significant (*p* < 0.05) change is indicated (*). Cells were also transfected with siHOTTIP or siHOTAIR and GDF15 protein was analyzed by Western blots. The 1457 value for siHOTTIP (C) includes some genes detected by more than one probe (also see Figure 4A).

Regulation of gene expression by HOTTIP was investigated by comparing Panc1 cells transfected with scrambled siRNA with those that were transfected with siHOTTIP in Panc1 cells followed by analysis of gene expression using an Illumina Human V.3 HT12 beadchip array [12]. Transfection of cells with siHOTTIP resulted in increased expression of 514 (HOTTIP-repressed genes) and decreased expression of 757 genes (HOTTIP-enhanced genes) (Figure 3A). Gene ontology enrichment analysis demonstrated that HOTTIP-regulated genes could be classified into several categories (Figure 3B), including those associated with the functions of HOTTIP in cell growth, survival and migration (Figures 1 and 2). Figure 3C illustrates that among the 1271 genes regulated by HOTTIP and 1006 genes regulated by HOTAIR [12], there were only 109 genes (<5%) commonly regulated by both lncRNAs. Among the 109 commonly regulated genes, 87 genes were decreased and 22 were increased after transfecting cells with siHOTTIP or siHOTAIR; however, with a cut-off of 2-fold the number of common genes regulated by both lncRNAs was only 13 (decreased) and 2 (increased). Growth differentiation factor 15 (GDF15) is an example of the differences between HOTTIP- and HOTAIR-regulated genes; in Panc1 cells knockdown of HOTAIR induced expression of GDF15, whereas siHOTTIP decreased expression of both GDF15 mRNA and protein (Figure 3D), and induction of GDF15 protein after HOTAIR knockdown was consistent with previous studies [12].

**Figure 4:**
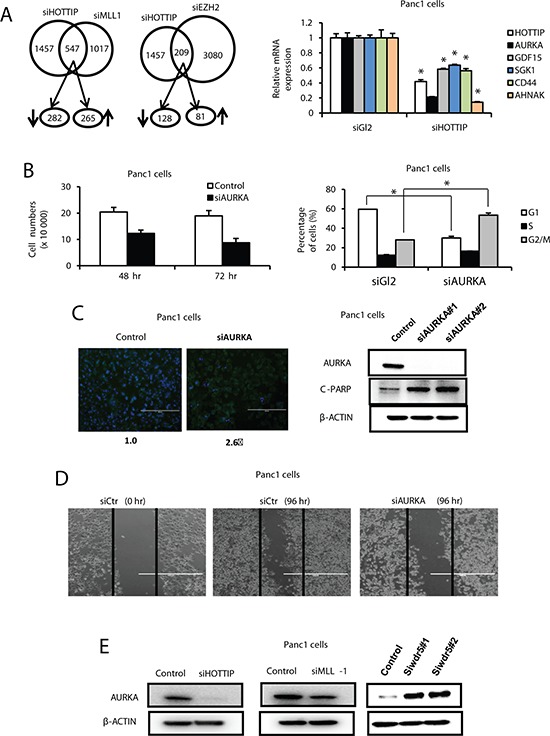
Interaction between HOTTIP, WDR5 and MLL1 on gene expression in Panc1 cells **(A)** Cells were transfected with siHOTTIP, siMLL, siEZH2 or siCtrl, and analyzed by microarrays for changes in gene expression. Regulation of the same genes is illustrated in the Venn diagram. Panc1 cells were transfected with siHOTTIP or siCtrl and genes coregulated by HOTTIP or MLL1 were analyzed by real time PCR. **(B)** Panc1 cells were transfected with siAURKA or siCtrl, and effects on cell growth and cell cycle progression were determined as outlined in the Materials and Methods. **(C)** Effects of Annexin V staining and PARP cleavage and **(D)** cell migration in a scratch assay were determined as Materials and Methods. **(E)** Panc1 cells were transfected with siHOTTIP, siMLL1 and siWDR5 or siCtrl and analyzed by Western blots as outlined in the Materials and Methods. Results are expressed as means ± SD for 3 replicated determinations, and significant (*p* < 0.05) change is indicated (*).

Since HOTTIP associates with the PRC2 and WDR5/MLL1 chromatin-modifying complexes [15], we compared the overlap in genes regulated by HOTTIP or these complexes after transfection of Panc1 cells with siHOTTIP, siMLL1 and siEZH2 followed by microarray analysis of changes in gene expression (Figure 4A). The results suggest that both MLL1 and HOTTIP or EZH2 and HOTTIP coregulate expression of 547 and 209 genes, respectively, and we further investigated genes commonly regulated by MLL1 and HOTTIP. Results in Figure 4B confirm that for several genes that contribute to enhanced carcinogenesis including *AURKA*, *AHNAK*, *GDF15*, *SGK1* and *CD44* [17–22], knockdown of HOTTIP resulted in decreased expression of these genes. Since AURKA (Aurora-A kinase) plays an important multifunctional role in pancreatic cancer [19–21], we further investigated the function and regulation of AURKA in pancreatic cancer cells. Results in Figure 4B show that knockdown of AURKA decreased Panc1 cell growth, and this was accompanied by a dramatic decrease in the percentage of cells in G_0_/G_1_ and an increase in cells in S and G_2_/M phase. Transfection of Panc1 cells with siAURKA also induced Annexin V staining and PARP cleavage (Figure 4C) and inhibited Panc1 cell migration in a scratch assay (Figure 4D). Transfection of Panc1 cells with siHOTTIP and siAURKA induced similar functional changes and this included inhibition of cell growth, induction of apoptosis, and decreased migration. However, the effects of siHOTTIP and siAURKA on cell cycle progression were different. We also observed that transfection of Panc1 cells with siHOTTIP and siMLL1 decreased expression of AURKA protein; however, transfection of cells with oligonucleotides that knockdown WDR5 (siWDR5) increased AURKA protein levels in Panc1 cells (Figure 4D). This indicates that the effects of HOTTIP/MLL1 on enhanced *AURKA* gene expression are independent of WDR5, and the coregulation of genes by HOTTIP and other MLL1-associated chromatin-modifying complexes is currently being investigated.

**Figure 5:**
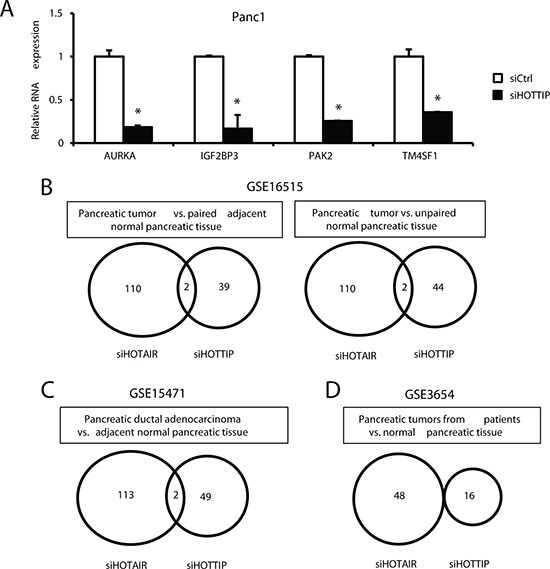
Inverse correlation of genes decreased by siHOTTIP and siHOTAIR and increased in pancreatic tumors **(A)** Panc1 cells were transfected with siHOTTIP, and expression of 9 selected genes upregulated in pancreatic tumors (GSE16515) was determined by real time PCR as outlined in the Materials and Methods. Overlap of expression of common genes induced in human tumors in GSE1655 **(B)**, GSE15471 **(C)** and GSE3654 **(D)** data sets and decreased in Panc1 cells transfected with siHOTTIP or siHOTAIR [12].

We also compared the inverse expression of genes in Panc1 cells transfected with siHOTTIP with those overexpressed in pancreatic tumors compared to paired adjacent normal tissue (GSE16515) and observed 39 genes that were inversely regulated (Supplementary Table 3). Figure 5A summarizes the effects of siHOTTIP on expression of 4 genes upregulated in tumors (GSE16515) and decreased after knockdown of HOTTIP in Panc1 cells. Examination of gene expression comparisons from GSE16515 (pancreatic tumor vs. paired adjacent normal tissue and unpaired normal pancreatic tissue) (Figure 5B), GSE15471 (tumor vs. adjacent normal tissue) (Figure 5C), and GSE3654 (tumor vs. normal pancreatic tissue) (Figure 5D) showed that 39, 44, 49 and 16 genes upregulated in tumors were downregulated in Panc1 cells transfected with siHOTTiP. In contrast, only a few (0–6 genes in the data sets) inversely related genes were downregulated in tumors and increased in Panc1 cells transfected with siHOTTIP and these were not included in the comparison with siHOTAIR results. Results in Figures 5B, and 5D show the number of common genes regulated in the four human tumor data sets and decreased in Panc1 cells after transfection with siHOTTIP or siHOTAIR [12] and the overlap of the common siHOTTIP/siHOTAIR genes. We observed a range of 0–2 overlapping genes in the four data sets. These results further emphasize that although both HOTTIP and HOTAIR have similar functions in pancreatic cancer, they regulate very different sets of genes.

### HOTTIP regulation of specific HOX genes

**Figure 6:**
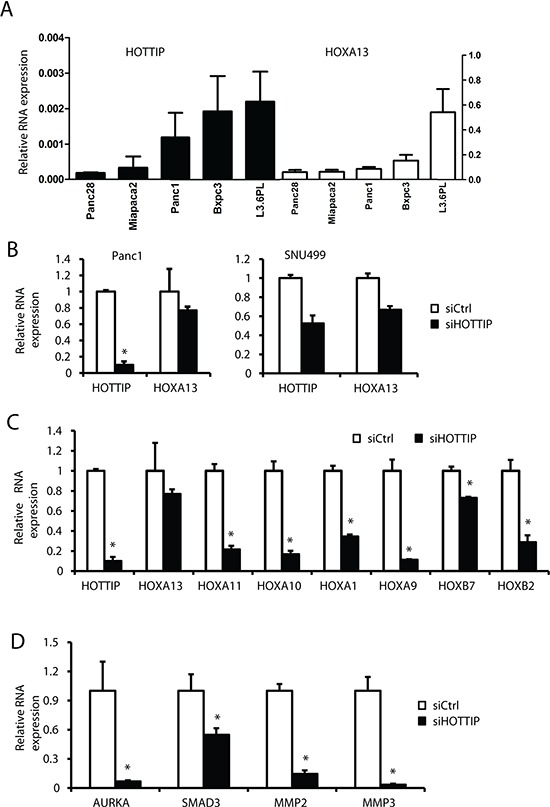
Coregulation of HOTTIP and *HOX* genes in pancreatic cancer cell lines **(A)** The relative expression of HOTTIP and HOXA13 compared to TBP mRNA in several pancreatic cancer cell lines was determined by real time PCR. **(B)** Panc1 and SNU499 cells were transfected with siHOTTIP and expression of HOXA13 was determined by real time PCR as outlined in the Materials and Methods. Panc1 cells were transfected with siHOTTIP and expression of several HOX mRNAs **(C)** and MMP/AURKA and SMAD3 mRNA **(D)** were determined by real time PCR as outlined in the Materials and Methods. Results are expressed as means ± SD for 3 replicated determinations, and significant (*p* < 0.05) change is indicated (*).

Previous studies in foreskin fibroblasts show that HOTTIP knockdown decreases expression of 5′ *HOXA* genes, particularly *HOXA13* [15] and in liver cancer cells and tumors, there was a parallel expression of HOTTIP and HOXA13 and siHOTTIP decreased HOXA13 mRNA levels [16]. HOXA13 is more highly expressed than HOTTIP by over 2 orders of magnitude in all of the pancreatic cancer cell lines (Figure 6A); however, despite these differences in the magnitude of expression, there was a correlation between expression of HOTTIP and HOXA13 in most of these cell lines. However, in Panc1 cells transfected with siHOTTIP, there was only a slight decrease in HOXA13 expression, whereas siHOTTIP decreased HOXA13 in SNU-499 liver cancer cells (Figure 6B) and this was consistent with previous studies in liver cancer cell lines [16]. It has previously been reported that some *HOX* genes are overexpressed in pancreatic tumors and they include *HOXA10*, *HOXB7* and *HOXB2* [23–27], and transfection of Panc1 cells with siHOTTIP slightly decreased expression of *HOXB7* but significantly decreased *HOXA10* (>80%) and *HOXB2* (>60%). Transfection with siHOTTIP also decreased mRNA levels of HOXA11 (>75%), HOXA9 (>80%) and HOXA1 (>60%), whereas siHOTTIP decreased expression of HOXB7 by <25% (Figure 6C). Thus, HOTTIP regulates expression of several *HOX* genes in pancreatic cancer cells but in contrast to liver cancer cells, HOTTIP does not regulate expression of *HOXA13*. A recent study showed that HOXA10 expression in pancreatic cancer cells was associated with regulation of matrix metalloproteinase 3 (MMP-3) [24] and in Panc1 cells transfected with siHOTTIP, we also observed decreased expression of HOXA10 and MMP-3 (Figure 6C and 6D). We also observed that SMAD3, a negative prognostic factor for pancreatic cancer patients and gene that promotes epithelial-mesenchymal transition [28] was also decreased after HOTTIP knockdown (Figure 6D). It has also been reported that HOXA11 regulates MMP-2 expression [29] and transfection with siHOTTIP also decreased both HOXA11 and MMP-2 (Figure 6C and 6D), suggesting that HOTTIP regulation of HOXA10 and HOXA11 and their downstream genes contribute to the oncogenic role of HOTTIP in pancreatic cancer cell migration/invasion.

## DISCUSSION

Rinn and coworkers have identified 231 non-coding RNAs associated with human *HOX* gene loci and these RNAs are spatially expressed and sequence-specific [7]. HOTAIR was the first HOX-associated lncRNA that was characterized and was initially identified as a scaffold RNA associated with the chromatin-modifying PRC2 complex and the H3K27me3 histone mark which is associated with gene suppression. Subsequent studies showed that HOTAIR directly interacted with both the PRC2 and LSD1/REST/coREST repressor complexes [7–9], and in multiple tumor and cancer cell lines, HOTAIR-regulated gene expression enhances tumorigenesis [8–14]. HOTAIRm2, Mistral, HOTTIP and more recently linc-HOXA1 are other HOX-associated lncRNAs that have been investigated [7–9, 30–32] and linc-HOXA1 represses expression of HOXA1 in combination with the cofactor PURB [32].

The pro-oncogenic functions and negative prognostic significance of HOTAIR have been reported for several cancers including pancreatic cancer, whereas with the exception of one paper on HOTTIP in liver tumors and cells [16], the expression and functions of other HOX-associated lncRNAs in cancer cell lines have not been extensively investigated. In liver cancer cell lines and tumors, HOTTIP is closely associated with expression of HOXA13 and knockdown of HOTTIP decreases expression of HOXA13 [16]. In the present study, we investigated the expression and function of HOTTIP in pancreatic cancer cells and compared the results to that observed in previous studies on HOTAIR in pancreatic cancer cells [12]. Results of knockdown and overexpression studies show that HOTTIP has functions comparable to that described for HOTAIR and plays a role in pancreatic cancer cell proliferation, survival and migration/invasion (Figures 1 and 2). However, a comparison of the genes regulated by HOTTIP and HOTAIR in Panc1 cells after knockdown by RNA interference showed that there was minimal gene overlap (Figure 3C), even though the pro-oncogenic functions of HOTTIP and HOTAIR are comparable. We also examined the overlap of genes overexpressed in publically available pancreatic tumor data sets and genes downregulated in Panc1 cells transfected with siHOTTIP (Figure 5). Among the human data sets (GSE16515, GSE15471 and GSE 3654), the number of overexpressed genes in tumors and genes downregulated by siHOTTIP that were in common varied among the data sets (16–49 genes in common) and similar variability (48–113 genes in common) was observed with genes downregulated by siHOTAIR in Panc1 cells [12]. Some of these differences in the number of common genes may be due to the sensitivity and composition of the different arrays that were used in these studies. However, the most striking observation was the minimal overlap between the genes regulated by HOTTIP vs. HOTAIR, further confirming the independent pro-oncogenic functions and gene regulation by these two lncRNAs in pancreatic cancer.

Since HOTTIP interacts with both the PRC2 and MLL1/WDR5 chromatin-modifying complexes [15], we also investigated by RNAi and microarrays, the overlap of genes coregulated by HOTTIP and MLL1/WDR5 (siHOTTIP/siMLL1) and by HOTTIP and PRC2 (siHOTTIP/siEZH2) (Figure 4A). Although both HOTTIP and MLL1 coregulated several genes in common, it was apparent that <40% of all HOTTIP-regulated genes were coregulated by HOTTIP and the two complexes. Moreover, among several genes that were decreased in Panc1 cells after transfection with siHOTTIP (Figure 4B), we observed that AURKA was coregulated by HOTTIP and MLL1 but not HOTTIP and WDR5 (Figure 4E) which has been reported to bind directly to HOTTIP [15]. Interestingly, knockdown of WDR5 increased AURKA protein levels, whereas siHOTTIP and siMLL1 decreased AURKA protein (Figure 4E). These results suggest regulation of gene expression by HOTTIP in pancreatic cancer cells involves interaction with complexes in addition to PRC2 and MLL1/WDR5, and this includes MLL1 complexes independent of WDR5. These interactions are currently being investigated.

Our results also showed that HOTTIP regulated expression of multiple *HOX* genes in pancreatic cancer cell cells (Figure 6C) but, in contrast to results in liver cancer cell lines [16], HOTTIP did not regulate expression of HOXA13 (Figure 6B). Previous studies indicated that HOXA10 and HOXA11 regulate expression of MMP-3 and MMP-2, respectively [24, 29], and both MMPs contribute to migration/invasion of pancreatic cancer cells [24, 33–35]. Figures 6C and 6D show that siHOTTIP decreased both HOXA10 and HOXA11 and this was paralleled by decreased expression of MMP-3 and MMP-2, demonstrating that HOTTIP regulates specific *HOX* genes that play a role in the migration/invasion of pancreatic cancer cells. Thus, HOTTIP functions in pancreatic cancer cells are due, in part, to regulation of some *HOX* genes but not *HOXA13* as previously observed in liver cancer cells [16]. In addition, the HOX gene targeted by in HOTTIP in pancreatic cancer cells are different from those regulated by HOTTIP in primary human fibroblasts [7]. Results of our study demonstrate a novel pro-oncogenic role for HOTTIP in pancreatic cancer cells, and we are currently investigating HOTTIP expression in tumors and both the *HOX*-dependent and -independent pro-oncogenic functions of HOTTIP in pancreatic and other cancer cell lines. Current studies are focused on the regulation of HOTTIP expression and the discovery of agents that target this lncRNA.

## MATERIALS AND METHODS

### Cell lines, reagents, and antibodies

Panc28 cells were a generous gift from Dr. Paul Chiao (University of Texas MD Anderson Cancer Center, Houston, TX), and the L3.6pL cell line was kindly provided by I. J. Fidler (University of Texas MD Anderson Cancer Center). Panc1, ASPC1, BxPC3, MiaPaCa2 cells were obtained from the American Type Culture Collection (Manassas, VA) and HPDE cells were provided by Dr. Ming Sound Tsao (Ontario Cancer Institute, Toronto, Canada). Panc1, L3.6pL, Panc28 and MiaPaCa2 cells were maintained in Dulbecco's modified Eagle medium (DMEM)-Ham's F-12 nutrient mixture (Sigma-Aldrich, St. Louis, MO) with phenol red supplemented with 0.22% sodium bicarbonate, 5% fetal bovine serum (FBS), and 10 ml/liter 100X antibiotic/antimycotic solution (Sigma-Aldrich). BxPC3 cells were maintained in RPMI-1640 medium (Sigma-Aldrich, St. Louis, MO) with phenol red supplemented with 0.15% sodium bicarbonate, 0.24 HEPES, 0.011% sodium pyruvate, 0.45% glucose, 10% FBS and 10 ml/liter 100X antibiotic/antimycotic solution (Sigma-Aldrich). Cells were grown in 150-cm^2^ culture plates in an air-CO_2_ (95:5) atmosphere at 37°C and passaged approximately every 3 to 5 days. Cleaved PARP (D214) antibodies were purchased from Cell Signaling Technology (Danvers, MA). β-Actin (A1978) was from Sigma-Aldrich; GDF15 (sc-377195) was from Santa Cruz Technology (Dallas, Texas) and Aurora A antibody (A300–071A) was from Bethyl Laboratories Inc. (Montgomery, TX). Chemiluminescence reagents (Immobilon Western) for Western blot imaging were purchased from Millipore (Billerica, MA). Lipofectamine 2000 was purchased from Invitrogen (Carlsbad, CA).

### RNA interference and plasmid transfection

Small interfering RNAs (siRNAs) for HOTTIP, MLL, WDR5, AURKA, and a non-specific control (Ctrl) were purchased from Sigma-Aldrich. The siRNA complexes used in this study are listed in Supplementary Table 1. Panc1 and L3.6pL cells were seeded (1 × 10^5^ per well) in 6-well plates in DMEM-Ham's F-12 medium supplemented with 2.5% charcoal-stripped FBS without antibiotic and left to attach for 1 day. Knockdown by RNA interference (RNAi) with siCtrl as a control was performed using Lipofectamine 2000 transfection reagent as per the manufacturer's instructions. Full length HOTTIP in pcDNA3.1+ was kindly provided by Dr. Howard Y. Chang (Stanford University, Stanford, CA) [15]. Panc28 cells were seeded (1 × 10^5^ per well) in 6-well plates n DMEM-Ham's F-12 medium supplemented with 2.5% charcoal-stripped FBS without antibiotic and left to attach for 1 day. PcDNA3.1-HOTTIP along with pcDNA3.1+ plasmid as a control was performed using Lipofectamine 2000 transfection reagent as per the manufacturer's instructions.

### Real time-PCR

Total RNA was isolated using the mirVana miRNA isolation kit (Ambion, Austin, TX) according to the manufacturer's protocol. RNA was eluted with 100 μl of RNase-free water and stored at –80°C. Real-time (RT)-PCR was carried out using iTaq Universal SYBR Green One-step Kit (BioRad). The primers used are listed in Supplementary Table 2. The housekeeping TATA-binding protein (TBP) mRNA was use as a control for comparing relative expression of RNAs.

### Western blot analysis

Pancreatic cancer cells were seeded in 6-well plates using 2.5% DMEM-Ham's F-12 medium, and after 24 hr, Western blot analysis of whole-cell lysates was performed essentially as described previously [12].

### Xenograft study

Female athymic nude mice, 4 to 6 weeks old, were purchased from Harlan Laboratories (Houston, TX). L3.6pL cells in culture were transfected with 100 nM siHOTTIP or siCT using Lipofectamine 2000. After 48 hr, cells were collected and 1 × 10^6^ cells in matrigel (1:1 ratio) were injected into either side of the flank area of female nude mice (Harlan). Tumor volumes were measured (0.5 × length × width^2^) throughout the study and after 10 days, the mice were sacrificed and tumor weights were determined. Tumor volumes and weights were determined in mice from the siHOTTIP (5 mice) or siCT (5 mice) groups, and siHOTTIP levels were determined by real time PCR. Research involving animal experimentation was reviewed and approved by the Texas A&M University Institutional Animal Care and Use Committee.

### Cell proliferation, death, and cycle analysis

Panc1 and L3.6pL cells were seeded in 12-well plates and permitted to attach for 24 hr, and then cells were transfected with 100 nM siRNA control or different siRNAs using Lipofectamine 2000 (Invitrogen, Grand Island, NY). Cells were trypsinized and counted at the indicated times using a Coulter Z1 cell counter (Beckman Coulter, Fullerton, CA). For cell cycle analysis, cells were stained with propidium iodide solution (50 μg/ml) and were analyzed by a FACSCalibur flow cytometer 24 hr after transfection. Apoptosis was detected using a fluorescein isothiocyanate (FITC) Annexin V staining kit (Life Technologies, Grand Island, NY) followed by fluorescence-activated cell sorter (FACS) analysis according to the manufacturer's protocol.

### Transwell migration and scratch assays

Panc1 and L3.6pL cells were first transfected with siRNA for 24 hr, then added to the upper chamber of a transwell chamber in duplicate and allowed to migrate into the lower chamber containing Hams F12 media with 20% FBS by incubating for 24 hr at 5% CO_2_ at 37°C. Cells migrating to the outer side of the upper chamber were fixed, stained and counted, and cell migration was also determined using a scratch assay. For the scratch assay, cells were first seeded in 6-well plate for 24 hr, and then a scratch through the central axis of the plate was gently made using a sterile pipette tip. Cells were transfected with siHOTTIP or siCtrl, media was changed after 6 hr, and migration of the cells into the scratch was observed after 48, 72 and 96 hr. Three replicates were obtained for each time point.

### Microarray analysis

Total RNA was extracted from Panc1 cells by using a mirVanaTM miRNA Isolation Labeling Kit (Ambion Inc.). The total RNA was quantified by using a Nanodrop ND-1000 spectrophotometer (NanoDrop Technology). The total RNA samples with adequate RNA quality index (>7) were used for microarray analysis; 700 ng of total RNA was used for labeling and hybridization on HumanHT-12 v4 expression beadchip (Illumina, Inc.) according to the manufacturer's protocols. After the beadchips were scanned with a BeadArray Reader (Illumina), the microarray data were normalized using the quantile normalization method in the Linear Models for Microarray Data (LIMMA) package in the R language (http://www.r-project.org). BRB-ArrayTools were primarily used for statistical analysis of gene expression data, and the Student's *t* test was applied to identify the genes significantly different between 2 groups when compared. Differentially expressed genes were identified using > 1.5 or 2 fold change cut off. Gene ontology enrichment analysis was carried out using David Functional Annotation Resources 6.7 (http://david.abcc.ncifcrf.gov/). Data for gene expression study of pancreatic ductal adenocarcinoma were downloaded from Gene Expression Omnibus (GEO, NCBI) (http://www.ncbi.nlm.nih.gov/geoprofiles/).

### Comparison of gene expression changes from lncRNA knockdown to a gene expression database

A rank-based nonparametric analysis strategy called the Running Fisher's algorithm and implemented within the NextBio database (http://www.nextbio.com/) environment was used to identify gene expression comparisons (biosets) which have statistically significant positive or negative correlation to the genes regulated by siHOTTIP. The Running Fisher's algorithm computes statistical significance of similarity between ranked fold-change values of two gene lists using a Fisher's exact test [36]. After exporting the analysis, the list of correlated biosets were filtered to identify those that examined gene expression changes in pancreatic cancers.

### Statistical analysis

Statistical significance of differences between the treatment groups was determined by an analysis of variance and/or Student's *t* test, and levels of probability were noted. At least 3 repeated experiments were determined for each data points and results are expressed as means ± SD.

## SUPPLEMENTARY TABLES


