# Supplementary material for: The long non-coding RNA HOTTIP enhances pancreatic cancer cell proliferation, survival and migration
Source: Oncotarget. 2015 Mar 25;6(13):10840–52. doi: 10.18632/oncotarget.3450 (PMC4484423; doi:10.18632/oncotarget.3450)
Supplement: Supplementary file 1 [file oncotarget-06-10840-s001.pdf]

SUPPLEMENTARY TABLES

Supplementary Table S1: The siRNA complexes used in this study

Name	Sequence
siGL2	CGU ACG CGG AAU ACU UCG A
siHOTTIP	GCACAGAGAUAAUGGCAAAUU
siMLL	SASI_Hs01_00090459
siWDR5#1	SASI_Hs01_00046875
siWDR5#2	SASI_Hs01_00046876
siAURKA#1	SASI_Hs01_00241476
siAURKA#2	SASI_Hs01_00241477

Supplementary Table S2: Primers used for Real Time-PCR

Name	Forward Primer	Reverse Primer
TBP	TGCACAGGAGCCAAGAGTGAA	CACATCACAGCTCCCCACCA
AHNAK	CTGAAGTGGTTCTGAGCGG	TCCACTCCATCTTCCGACTT
HOXA13	GGATATCAGCCACGACGAAT	ATTATCTGGGCAAAGCAACG
HOXA11	TGCCAAGTTGTACTTACTACGTC	GTTGGAGGAGTAGGAGTATGTCA
HOXB7	CGAGTTCCTTCAACATGCACT	TTTGCGGTCAGTTCCTGAGC
HOXA9	CTGTCCCACGCTTGACACTC	CTCCGCCGCTCTCATTCTC
HOXA10	TGGCTCACGGCAAAGAGTG	GCTGCGGCTAATCTCTAGGC
SMAD3	TGGACGCAGGTTCTCCAAAC	CCGGCTCGCAGTAGGTAAC
MMP3	GAGCTAAGTAAAGCCAGTGGA	GATATTTCTGAACAAGGTTCATCGT
HOTTIP	CCTAAAGCCACGCTTCTTTG	TGCAGGCTGGAGATCCTAGT
SGK1	GCGCTAACGTCTTTTCTGTCT	TGCTTCATGAAAGCGATGAG
GDF15	CTCCAGATTCCGAGAGTTGC	CACTTCTGGTGAGTATCC
MMP2	CCCACTGCGGTTTTCTCGAAT	CAAAGGGGTATCCATCGCCAT
CD44	TGCTACCAGAGACCAAGACA	CCCATGTGAGTGTCCATCTG
AURKA	GTCAAGTCCCCTGTCGGTT	AGTGAGACCCTCTAGCTGT
HOXA1	TCCTGGAATACCCCATACTTAGC	GCACGACTGGAAAGTTGTAATCC
IGF2BP3	ATTTACAGTGGGAGGTGCTG	GCAGTTTCCGAGTCAGTGTT
TM4SF1	TGGTTCTTTTCTGGCATCGT	AGAAAGCATCGCACATCGTT
PAK2	GCCAAAGAATTATTACAGCATCC	TGCTTCTTTAGCTGCCATGA

Supplementary Table S3: Common genes overexpressed in pancreatic tumors (GSE16515) and downregulated in Panc1 cells transfected with siHOTTIP

Gene	EntrezGene ID	Gene Description	GSE16515 Pancreatic tumor _vs_ paired adjacent normal pancreatic tissue (Study: Pancreatic tumor compared to normal pancreatic tissue)	HOTTIP regulated gene list
TM4SF1	4071	transmembrane 4 L six family member 1	4.19	–5.21
SMS	6611	spermine synthase	2.1	–4.8
IGF2BP3	10643	insulin–like growth factor 2 mRNA binding protein 3	8.48	–4.38
NCEH1	57552	neutral cholesterol ester hydrolase 1	2.45	–4.06
UBASH3B	84959	ubiquitin associated and SH3 domain containing B	2.37	–4.02
PLAU	5328	plasminogen activator, urokinase	6.39	–3.69
NT5E	4907	5′-nucleotidase, ecto (CD73)	2.94	–3.65
PAK2	5062	p21 protein (Cdc42/Rac)-activated kinase 2	2.22	–3.62
AURKA	6790	aurora kinase A	2.52	–3.56
LDLR	3949	low density lipoprotein receptor	2.38	–3.43
F2RL1	2150	coagulation factor II (thrombin) receptor-like 1	2.29	–3.38
SLC44A1	23446	solute carrier family 44, member 1	2.32	–3.34
ADORA2B	136	adenosine A2b receptor	3.45	–3.2
PNMA2	10687	paraneoplastic Ma antigen 2	2.02	–2.88
ALDOC	230	aldolase C, fructose-bisphosphate	2.27	–2.67
ERO1L	30001	ERO1-like (S. cerevisiae)	3.87	–2.52
ITGB1	3688	integrin, beta 1 (fibronectin receptor, beta polypeptide, antigen CD29 includes MDF2, MSK12)	2.11	–2.49

(*Continued*)

Gene	EntrezGene ID	Gene Description	GSE16515 Pancreatic tumor _vs_ paired adjacent normal pancreatic tissue (Study: Pancreatic tumor compared to normal pancreatic tissue)	HOTTIP regulated gene list
YWHAZ	7534	tyrosine 3-monooxygenase/ tryptophan 5-monooxygenase activation protein, zeta polypeptide	2.37	–2.42
RAB31	11031	RAB31, member RAS oncogene family	3.39	–2.4
PALLD	23022	palladin, cytoskeletal associated protein	3.09	–2.39
ELF4	2000	E74-like factor 4 (ets domain transcription factor)	2.63	–2.38
KIF11	3832	kinesin family member 11	3.12	–2.36
DPY19L1	23333	dpy-19-like 1 (C. elegans)	2.69	–2.35
SEMA4B	10509	sema domain, immunoglobulin domain (Ig), transmembrane domain (TM) and short cytoplasmic domain, (semaphorin) 4B	2.48	–2.32
PHLDA1	22822	pleckstrin homology-like domain, family A, member 1	2.45	–2.32
CD97	976	CD97 molecule	2.32	–2.31
SLC16A3	9123	solute carrier family 16, member 3 (monocarboxylic acid transporter 4)	8.25	–2.29
ECT2	1894	epithelial cell transforming sequence 2 oncogene	5.83	–2.28
TMEM189	387521	transmembrane protein 189	2.05	–2.28
CALML4	91860	calmodulin-like 4	2.74	–2.24
SDC1	6382	syndecan 1	6.28	–2.18
ITGB5	3693	integrin, beta 5	2.34	–2.12
CCNB1	891	cyclin B1	4.14	–2.09

(*Continued*)

Gene	EntrezGene ID	Gene Description	GSE16515 Pancreatic tumor _vs_ paired adjacent normal pancreatic tissue (Study: Pancreatic tumor compared to normal pancreatic tissue)	HOTTIP regulated gene list
SLC6A10P	386757	solute carrier family 6 (neurotransmitter transporter, creatine), member 10, pseudogene	2.64	–2.09
NUAK1	9891	NUAK family, SNF1-like kinase, 1	2.74	–2.04
CLIC1	1192	chloride intracellular channel 1	2.7	–2.04
ENO2	2026	enolase 2 (gamma, neuronal)	3.91	–2.01
MALL	7851	mal, T-cell differentiation protein-like	6.77	–2
KIF2C	11004	kinesin family member 2C	2.65	–2
